# Sequence of Changes in Maize Responding to Soil Water Deficit and Related Critical Thresholds

**DOI:** 10.3389/fpls.2018.00511

**Published:** 2018-05-01

**Authors:** Xueyan Ma, Qijin He, Guangsheng Zhou

**Affiliations:** ^1^Chinese Academy of Meteorological Sciences, Beijing, China; ^2^Tianjin Meteorological Bureau, Tianjin, China; ^3^College of Resources and Environmental Sciences, China Agricultural University, Beijing, China; ^4^Collaborative Innovation Center on Forecast Meteorological Disaster Warning and Assessment, Nanjing University of Information Science & Technology, Nanjing, China

**Keywords:** indicators, maize, sequence of changes, soil drought, soil moisture content, critical thresholds

## Abstract

The sequence of changes in crop responding to soil water deficit and related critical thresholds are essential for better drought damage classification and drought monitoring indicators. This study was aimed to investigate the critical thresholds of maize growth and physiological characteristics responding to changing soil water and to reveal the sequence of changes in maize responding to soil water deficit both in seedling and jointing stages based on 2-year’s maize field experiment responding to six initial soil water statuses conducted in 2013 and 2014. Normal distribution tolerance limits were newly adopted to identify critical thresholds of maize growth and physiological characteristics to a wide range of soil water status. The results showed that in both stages maize growth characteristics related to plant water status [stem moisture content (SMC) and leaf moisture content (LMC)], leaf gas exchange [net photosynthetic rate (Pn), transpiration rate (Tr), and stomatal conductance (Gs)], and leaf area were sensitive to soil water deficit, while biomass-related characteristics were less sensitive. Under the concurrent weather conditions and agronomic managements, the critical soil water thresholds in terms of relative soil moisture of 0–30 cm depth (RSM) of maize SMC, LMC, net Pn, Tr, Gs, and leaf area were 72, 65, 62, 60, 58, and 46%, respectively, in seedling stage, and 64, 64, 51, 53, 48, and 46%, respectively, in jointing stage. It indicated that there is a sequence of changes in maize responding to soil water deficit, i.e., their response sequences as soil water deficit intensified: SMC ≥ LMC > leaf gas exchange > leaf area in both stages. This sequence of changes in maize responding to soil water deficit and related critical thresholds may be better indicators of damage classification and drought monitoring.

## Introduction

Crop growth is affected by a variety of abiotic factors, such as climate, cultivation, soil fertility, water efficiency, etc. (Meng et al., 2005; Guo et al., 2010; Alkaisi et al., 2015; Testa et al., 2016). Many of the impacts of climate and agronomical are felt by crops through the filter of soil moisture dynamics, because crops get their water from soil. Therefore, soil water deficit has the most significant impact on crops (Denmead and Shaw, 1962; Porporato et al., 2001). It restricts the growth, development, and yield of crops worldwide, consequently making losses exceeding the total caused by all other adverse factors (Ray et al., 2002; Neumann, 2008; Zivcak et al., 2008; Farooq et al., 2009; Bajgain et al., 2016; Petrov et al., 2017). Extensive studies on plant responses to soil water deficit at various levels (i.e., gene, cell, organ, individual) have been reported, serving a great purpose in understanding plant’s response mechanisms to soil water deficit (Blum, 1996; Tardieu, 1996; Lawlor, 2002; Yordanov et al., 2000; Neumann, 2008; Ghannoum, 2009; Anjum et al., 2011; Pinheiro and Chaves, 2011). However, majority of these studies focused on qualitative impacts of soil water deficit with certain intensities on crop growth, based on experiments with very limited amounts of soil water levels.

In fact, only when soil water below a critical point that would exert significant impacts on crops (Blum, 1996; Sadras and Milroy, 1996; Ray et al., 2002; Streck, 2004; Novák, 2009; Gholipoor et al., 2012; Ramadas and Govindaraju, 2015; Esmaeilzade–Moridani et al., 2015). Any plant constituent and physiological process can be altered sequentially if soil water deficit is severe enough and lasts long enough, but they probably respond to different critical soil water status, namely, they have different sensitivities to soil water deficit (Hsiao, 1973; Hsiao et al., 1976; Andersen et al., 2002; Wu et al., 2011b). The sequence of changes in crop responding to soil water deficit and related critical thresholds are essential for identifying the extent of crop damage and improving drought prevention and resistance capabilities, which have been seldom drawn attention yet (Andersen et al., 2002). Hsiao et al. (1976) had reviewed extensive studies and evolved the sequence of changes of plant physiology and metabolism occurred in minutes or hours responding to water stress. Some studies attempted to identify critical soil water status of plant physiological and growth traits, but they were generally confined to certain aspects of plant growth, such as leaf expansion and leaf transpiration, lacking a comprehensive comparison among various levels of growth characteristics that reflect the plant growth status in response to water stress (Nable et al., 1999; Ray et al., 2002; Casadebaig et al., 2008; Heinemann et al., 2011).

The critical soil water thresholds were usually obtained either by direct observation of the critical points when the plant characteristics varied significantly or by calculating the stagnation points of the regression models between crop growth characteristics and their soil water status (Sadras and Milroy, 1996; Xu et al., 2010). Typical regression models included logistic regression model (Soltani et al., 2000; Toms and Villard, 2015), negative exponential regression model (Sadras and Milroy, 1996), quadratic polynomial model (Xu et al., 2010), plateau regression model (Nable et al., 1999; Wang et al., 2008; Yan et al., 2010; Wu et al., 2011a; Meir et al., 2015), and linear spline model (Soltani et al., 2000). However, the observed samples were usually quite limited due to the restriction of simulation experiments in the amounts and ranges of soil water gradients, which may not include the critical thresholds or be unable to establish a regression model well enough to accurately identify the critical thresholds.

Therefore, investigations on quantitative responses of various crop growth characteristics to a wide range of soil water conditions were of great necessity in order to accurately identify the sequence of changes in plant responding to soil water deficit and related critical soil water status, which would contribute to indicate the occurrence and development of drought and serve to more timely drought monitoring (Thompson et al., 2007).

Maize (*Zea Mays* L.) is the leading crop worldwide and pivotal to current and future global food security (Anjum et al., 2016). Usually, maize predominates in hot, arid regions which are prone to frequent drought and would likely be exacerbated by global climate change (Ghannoum, 2009). In this study, a 2-years’ maize field experiment responding to soil water deficit were conducted, the aims were to (1) reveal the sequence of changes in maize responding to soil water deficit, and (2) identify critical soil water thresholds related to the sequence of changes in maize.

## Materials and Methods

### Site Descriptions

The research site was at Gucheng Agrometeorological Experimental Station of China Meteorological Administration. It was located in Dingxing County, Baoding City, Hebei Province, China (39° 08′ N, 115° 40′ E), belonging to the maize planting zone across wide Northern China. The station was equipped with an auto-rain-shelter, which covered an area of 750 m^2^ and was divided into 42 trial plots, each was 2 m-wide × 4 m-long and was isolated by 3 m-deep concrete walls to prevent soil water exchange horizontally. The site has a typical cinnamon soil, containing 13.67 g kg^-1^ organic C, 0.87 g kg^-1^ total N, 25.76 mg kg^-1^ available P, and 118.55 mg kg^-1^ available K. The soil bulk density is 1.37 g cm^-3^, and pH is 8.1. The average field capacity and wilting point is 0.23 and 0.07 g g^-1^, respectively (Fang et al., 2013).

### Experimental Design

A 2-years’ maize field experiment responding to six initial soil water statuses was conducted in 2013 and 2014. *Zhengdan 958*, the most popular maize genotype in China was used in both years’ experiments. In 2013, the maize was sown on 27 June with a 50 cm line spacing and a 30 cm row spacing, giving a plant density of 6.5 plants m^-2^. In 2014, the maize was sown on 24 June with a 50 cm line spacing and a 25 cm row spacing, giving a plant density of 8.0 plants m^-2^. Diammonium Phosphate fertilizer was applied at 300 kg ha^-1^ before sowing each year, equal to the fertilization level of local field. All other agronomic managements were identical to the local field in both years.

A completely randomized block design with three replicate plots was applied in both years. Irrigation was performed every other day to ensure the relative soil moisture of 0–50 cm depth of each trial plot is above 65%, maintaining normal growth of maize plants before they expanded the 7th leaf (July 24th, 2013) and the 3rd leaf (July 2nd, 2014), respectively (Guo et al., 2001; Xiao et al., 2011). Then, six different irrigations were performed. In 2013, the irrigation amounts (named 1st–6th treatments) were 100, 80, 60, 40, 25, and 15 mm, respectively, equivalent to 125, 100, 75, 50, 30, and 20% of the local average precipitation in late July (80 mm), respectively. In 2014, the irrigation amounts (named 1st–6th treatments) were 150, 120, 90, 60, 30, and 10 mm, respectively, equivalent to 100, 80, 60, 40, 20, and 7% of the local average precipitation in July (150 mm), respectively. No extra irrigation was performed thereafter. Precipitation was blocked completely by the auto-rain-shelter during the entire growth period.

### Measurements

We made one observation in the seedling stage on July 9th, 2014, and two observations in the jointing stage on July 31st, 2013, and August 8th, 2013, respectively. Eleven indicators involving plant water status, leaf gas exchange, morphology and biomass of maize were measured as candidates to investigate their responses to soil water deficit. They are moisture content of the stem and the leaf (LMC and SMC), net photosynthetic rate (Pn), transpiration rate (Tr), stomatal conductance (Gs), leaf area (LA), root–shoot ratio (R/S), dry biomass of the leaf, the stem, and the root (LDB, SDB, and RDB), and total dry biomass of the plant (TB).

#### Leaf Gas Exchange

Measurements on leaf gas exchange were performed with a Li-6400 portable photosynthesis system equipped with a standard leaf chamber (LI-COR, Lincoln, NE, United States). The net Pn, Tr, and Gs were measured on the youngest fully expanded leaf under natural light between 9:00 am and 11:00 am in clear weather.

#### Leaf Area (LA)

The length (*L*_i_) and width (the widest part of the leaf, *D*_i_) of every fully expanded leaf on the sample plants were measured. The length of inadequately expanded leaf was measured based on its exposed part from the last leaf, and its width was estimated based on its original shape without being spread out. LA (*m*^2^) of individual maize plant was obtained by Eqn. (1):

(1) $$LA=\sum_{i\text{=}1}^{n}L_{i}\times D_{i}\times k$$

Where *k* (=0.75) is the shape factor (Francis et al., 1969).

#### Biomass and Plant Water Status

The fresh mass of leaf and stem was weighed, while the root was rinsed. They were separately put into kraft bags and dried in an oven at 80°C for more than 24 h until their weights were constant. Then, biomass of leaf, stem, and root was weighed, respectively. Stem moisture content (SMC), leaf moisture content (LMC), and root/shoot ratio (R/S) were calculated according to the following formulas (Peuke et al., 2002):

SMC = (SFB - SB)/SFB × 100%

LMC = (LFB - LB)/LFB × 100%

R/S = RB/(LB + SB)

Where SFB, SB, LFB, LB, and RB were stem fresh biomass, stem dry biomass, leaf fresh mass, leaf dry biomass, and root dry biomass, respectively.

#### Soil Water Content

Soil water content was measured by oven-drying method. One sampling point was randomly selected between two rows of maize in a trial plot and thus three samples obtained from each treatment in total. Soil samples of every 10 cm were collected from each sampling point. The total depth was up to 50 cm in 2013, while it was 90 cm in 2014. The samples were weighed both before and after they were dried up in an oven at 105°C. The relative soil moisture of 0–30 cm depth (RSM) was used here to describe soil water status (Eqn. 2), because it was most closely related to maize growth characteristics among all these measured depths (Supplementary Tables S1–S3).

(2) $$RSM=\frac{\sum_{i\text{=}1}^{3}\left(FS_{i}-DS_{i}\right)/\sum_{i\text{=}1}^{3}DS_{i}}{FC}\times 100\%$$

Where *FS* and *DS* were fresh and dry weights of soil samples from certain layers; *I* was the number of soil layer (*i* = 1, 2, 3 refers to soil layer 0–10, 11–20, and 21–30 cm, respectively); *FC* was the field capacity (=0.23 g g^-1^).

#### Meteorological Data

Meteorological data including temperature, relative humidity, wind speed, and total radiation at 1 min interval was obtained from the automatic weather station of Gucheng Agrometeorological Experimental Station. Vapor pressure deficit (VPD) was calculated by temperature and relative humidity.

### Tipping Points of Maize Growth and Related Critical RSM Thresholds

The tipping point of plant growth characteristic is the very point when values of the characteristic start to deviate significantly from those with sufficient water supply, resulting from a decline in soil water content below a critical level (Thompson et al., 2007; Czajkowski et al., 2009). Here, the method named as one-side upper and lower tolerance limits for normal population was newly adopted to identify the tipping point of each growth characteristic.

Tolerance limit refers to either of two quantities that specify the endpoints of a tolerance interval. A tolerance interval is an estimated interval within which at least a certain proportion *P* of the population falls at a given level of confidence γ (ISO 16269-6, 2005; Krishnamoorthy and Mathew, 2009; Young, 2014). Their rigorous statistical definitions could be found in Young (2014). ISO 16269-6 (2005) provides computational formulas of tolerance intervals and limits for different distribution populations. Given the sampling methods of the field experiment, formulas of one-side upper and lower tolerance limits for normal population with unknown variance and unknown mean were used here (Eqn. 3 and 4).

(3) $$U(X)\;\text{=}\;\bar{X}+k\times S$$

(4) $$L(X)\;\text{=}\;\bar{X}-k\times S$$

In the above, *U*(*X*) and *L*(*X*) are one-side upper and lower tolerance limits for normal population, respectively; 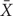 is the sample mean and *S* is the sample variance; *k* is the tolerance factor, varying with the sample size *n*, the confidence level *P*, and the γ percentile of the population included, which could refer to ISO 16269-6 (2005) or be calculated directly (Krishnamoorthy and Mathew, 2009).

To be specific, according to the results of Duncan multiple test, the growth characteristics from experimental treatments which had received larger amounts of irrigation while had no significant differences were thought to be still free of soil water deficit. Its tipping point was obtained by calculating the tolerance limit of the observed samples from these treatments. In terms of a growth characteristic which decreases as soil moisture drops below a certain level, its tipping point could be found by calculating the lower tolerance limit of these observed samples. It means that the characteristic would be unlikely lower than this tolerance lower limit when soil water is sufficient, while values of this characteristic would be probably lower than this limit once it was confronted with soil water stress. Accordingly, the tipping points of growth characteristics whose values would increase once confronted with water stress could be found by calculating the upper tolerance limits of observed samples that free of soil water deficit.

Since the formulas above could be used only when the samples normally distributed, we should first check the normality of the samples.

The tipping points of different growth characteristics were further quantified by their values of critical RSM thresholds, which were calculated in terms of the tipping points and the quadratic polynomial regression models between each growth characteristic and RSM (Eqn. 5).

(5) $$X\;\text{=}\;a\times RSM^{2}+b\times RSM+c$$

Where *X* is the observed values of a maize growth characteristic; *RSM* (%) is the relative soil moisture of 0–30 cm depth; *a, b*, and *c* are the fitting coefficients of the regression model.

### Statistical Analysis

One-way Multivariate Analysis of Variance (One-way MANOVA) was performed on RSM and each growth characteristic to assess their differences among treatments. Their means of each treatment were then compared by Duncan multiple test at 0.05 significance level. Factor analysis was used to extract a common factor from LMC and SMC to represent maize water status, and a common factor from net Pn, Gs, and Tr to represent leaf gas exchange, which would be subjected to exploratory path analysis to figure out how maize growth characteristics respond to soil water. Normality of the observed samples that would be subjected to the tolerant limits calculation was examined with Shapiro–Wilk method (Razali and Wah, 2011). All statistical analyses were performed by SPSS 17.0 software (SPSS Inc., Chicago, IL, United States), except that exploratory path analysis was performed by IBM SPSS AMOS 21.0 (SPSS Inc., Chicago, IL, United States). Figures were plotted by Origin 8.5 (OriginLab, United States). The critical RSM of each sensitive growth characteristic was calculated by Matlab software (Mathworks Matlab R2010b, United States). The data were shown as the mean ± standard deviation (mean ± SD).

## Results

### Changes in Maize Responding to Soil Water Deficit

#### Changes in Maize Responding to Soil Water Deficit in Seedling Stage

In 2014, the first observation was conducted on 9 July, i.e., 7 days after irrigation controls. Maize plants of six irrigation treatments were all in seedling stage. RSM was significantly different among treatments (**Tables 1, 2**). SMC of the 3rd to 6th treatments were significantly lower than those of the 1st and 2nd treatments; LMC of the 5th and 6th treatments were significantly lower than those of the 1st–4th treatments; net Pn, Tr, and Gs of the 4th–6th treatments were significantly lower than those of the 1st–3rd treatments; leaf area of the 6th treatment was significantly lower than those of the 1st–5th treatments; biomass related characteristics still not appeared any significant difference under current soil water status (**Tables 1, 2**). Therefore, growth characteristics related to plant water status (SMC and LMC), leaf gas exchange (net Pn, Tr, and Gs), and leaf area were more sensitive to soil water deficit among all these growth characteristics in the seedling stage.

**Table 1:** Significant levels of differences among treatments of RSM and maize growth characteristics.

Indicators	Units	July 09, 2014	July 31, 2013	August 08, 2013
		*P*	*P*	*P*
RSM	%	^∗∗∗^	^∗∗^	^∗∗^
Leaf moisture content	%	^∗∗∗^	^∗∗^	^∗^
Stem moisture content	%	^∗∗∗^	^∗∗^	^∗∗^
Root-shoot ratio	–	0.960	0.411	0.616
Leaf area	cm^2^ plant^-1^	^∗^	0.458	^∗∗^
Leaf dry mass	g plant^-1^	0.639	0.584	0.112
Stem dry mass	g plant^-1^	0.599	0.664	0.153
Root dry mass	g plant^-1^	0.978	0.122	0.318
Total dry mass	g plant^-1^	0.961	0.573	0.127
Net photosynthetic rate	μmol CO_2_ m^-2^s^-1^	^∗∗∗^	0.348	^∗∗∗^
Stomatal conductance	mol H_2_O m^-2^s^-1^	^∗∗∗^	0.134	^∗∗^
Transpiration rate	mmol H_2_O m^-2^s^-1^	^∗∗∗^	0.278	^∗∗^

*^∗∗∗^P < 0.001, ^∗∗^P < 0.01, ^∗^P < 0.05*.

**Table 2:** Duncan multiple test of RSM and sensitive growth characteristics of maize in seedling stage in 2014.

Treatments	RSM	LA	LMC	SMC	Pn	Tr	Gs
1	96.5 ± 1.0^a^	121.2 ± 24.6^a^	85.0 ± 0.4^a^	90.7 ± 0.3^a^	36.01 ± 3.31^a^	10.21 ± 1.31^a^	0.28 ± 0.05^a^
2	90.8 ± 1.4^b^	122.0 ± 16.1^a^	84.4 ± 0.6^a^	90.2 ± 0.4^ab^	32.46 ± 1.42^ab^	9.18 ± 0.42^a^	0.24 ± 0.02 ^ab^
3	83.1 ± 4.7^c^	107.9 ± 3.2^a^	84.1 ± 0.6^a^	89.7 ± 0.3^bc^	34.28 ± 0.64^a^	9.13 ± 0.11^a^	0.25 ± 0.01^a^
4	69.1 ± 2.6^d^	114.1 ± 2.4^a^	84.1 ± 0.9^a^	89.1 ± 0.4^c^	29.10 ± 0.57^bc^	7.79 ± 0.42^b^	0.20 ± 0.02^bc^
5	61.3 ± 4.5^e^	107.6 ± 5.6^a^	81.8 ± 0.4^b^	87.9 ± 0.2^d^	27.25 ± 0.90^c^	7.09 ± 0.36^b^	0.18 ± 0.02^c^
6	45.3 ± 1.1^f^	76.2 ± 3.8^b^	77.9 ± 1.8^c^	86.0 ± 0.4^e^	11.06 ± 2.52^d^	2.93 ± 0.51^c^	0.05 ± 0.02^d^

*Values marked with no same letter within a column denote significant differences (P < 0.05). RSM, relative soil moisture (%); LA, leaf area (cm^*2*^ plant^-*1*^); LMC, leaf moisture content (%); SMC, stem moisture content (%); Pn, net photosynthetic rate (μmol CO_*2*_ m^-*2*^s^-*1*^); Tr, transpiration rate (mmol H_*2*_O m^-*2*^s^-*1*^); Gs, stomatal conductance (mol H_*2*_O m^-*2*^s^-*1*^).*

#### Changes in Maize Responding to Soil Water Deficit in Jointing Stage

In 2013, the first observation was conducted on 31 July, 7 days after irrigation controls. Maize plants of six irrigation treatments were all in the jointing stage. RSM differed significantly among treatments (**Tables 1, 3**). Only stem and LMCs of the 6th treatment were significantly lower than those of the 1st–5th treatments (**Tables 1, 3**). On 8 August, i.e., the 14th day after irrigation controls, maize plants from the six treatments were still in the jointing stage. Differences in RSM among treatments were still significant (**Tables 1, 3**). LMC of the 5th and 6th treatments were much lower than that of the 1st treatment; SMC and Gs of the 4th–6th treatments were significantly lower than those of the 1st treatment; net Pn and Tr of the 5th and 6th treatments were significantly lower than those of the 1st–4th treatments; leaf area of the 5th and 6th treatments were significantly lower than those of the 1st and 2nd treatments; biomass-related characteristics were no apparent differences among treatments. Thus, growth characteristics related to plant water status (SMC and LMC), leaf gas exchange (net Pn, Tr, and Gs), and leaf area were more sensitive to soil water deficit among all these growth characteristics in jointing stage.

**Table 3:** Duncan multiple test of RSM and sensitive growth characteristics of maize in jointing stage in 2013.

Date	July, 31st			August, 8th						
Treatments	RSM	LMC	SMC	RSM	LA	LMC	SMC	Pn	Tr	Gs
1	93.9 ± 5.6^a^	84.0 ± 0.5^a^	91.0 ± 0.3^a^	68.9 ± 1.8^a^	1804.7 ± 471.8^a^	84.2 ± 1.1^a^	94.2 ± 0.09^a^	42.72 ± 1.76^a^	8.97 ± 0.73^a^	0.40 ± 0.08^a^
2	88.3 ± 11.0^ab^	83.9 ± 0.3^a^	91.3 ± 0.3^a^	66.1 ± 4.6^ab^	1710.4 ± 63.9^a^	83.3 ± 0.3^ab^	93.5 ± 0.8^ab^	37.27 ± 1.84^a^	7.64 ± 1.32^a^	0.31 ± 0.04^ab^
3	88.0 ± 3.4^ab^	83.8 ± 0.0^a^	91.5 ± 0.6^a^	65.5 ± 4.3^ab^	1403.6 ± 209.0^ab^	83.8 ± 0.6^ab^	93.8 ± 0.3^ab^	37.00 ± 3.96^a^	7.67 ± 1.57^a^	0.36 ± 0.07^ab^
4	80.3 ± 7.9^bc^	83.9 ± 0.2^a^	90.9 ± 0.3^a^	59.0 ± 7.0^bc^	1654.2 ± 70.5^a^	83.6 ± 0.4^ab^	92.7 ± 0.5^bc^	35.60 ± 0.64^a^	7.52 ± 0.52^a^	0.27 ± 0.03^bc^
5	69.3 ± 3.6^cd^	84.1 ± 0.2^a^	90.8 ± 0.6^a^	53.8 ± 3.5^cd^	1032.0 ± 90.5^bc^	82.6 ± 0.5^bc^	92.7 ± 0.4^bc^	28.50 ± 7.02^b^	5.34 ± 0.88^b^	0.19 ± 0.08^cd^
6	63.4 ± 3.1^d^	82.6 ± 0.3^b^	89.6 ± 0.6^b^	50.6 ± 4.0^d^	930.2 ± 191.7^c^	80.8 ± 1.2^c^	92.2 ± 0.3^c^	20.71 ± 3.94^c^	3.45 ± 1.18^b^	0.12 ± 0.03^d^

*Values marked with no same letter within a column denote significant differences (P < 0.05). RSM, relative soil moisture (%); LA, leaf area (cm^*2*^ plant^-*1*^); LMC, leaf moisture content (%); SMC, stem moisture content (%); Pn, net photosynthetic rate (μmol CO_*2*_ m^-*2*^s^-*1*^); Tr, transpiration rate (mmol H_*2*_O m^-*2*^s^-*1*^); Gs, stomatal conductance (mol H_*2*_O m^-*2*^s^-*1*^).*

### Effects of Soil Water Deficit on Sequence of Changes in Maize

The first common factors extracted from leaf and SMC based on the data of July 09, 2014, July 31, 2013, and August 08, 2013 account for 95.56, 85.64, and 82.57% of total variance, respectively. The first common factors extracted from net Pn, Gs, and Tr based on the data of July 09, 2014, July 31, 2013, and August 08, 2013 account for 97.94, 86.92, and 94.84% of total variance, respectively. These common factors were subjected to exploratory path analysis as representatives of plant water status and leaf gas exchange. An initial exploratory path diagram (Supplementary Figure S2a) was established to evaluate both the direct and indirect effects of soil water on plant water status, leaf gas exchange, leaf area, and plant total biomass. The maximum likelihood method was adopted to calculate the regression weight of each path and the squared multiple correlation (*R*^2^) of each dependent variate. Insignificant paths were deleted in order to attain the criteria of the goodness-of-fit indices. The final path models were shown in Supplementary Figures S2b–d, respectively. The model fit summary (Supplementary Table S4) showed that the path analysis model generally had a reasonable fit for all the observation data (Arbuckle, 2010). Although the effective paths were not identical among path models based on different data, the results generally showed that RSM had the most significant total effects on plant water status, less on leaf gas exchange and leaf area, and the least on plant total biomass; besides, its effects on plant water status were direct, while its effects on leaf area, leaf gas exchange, and plant total biomass were major indirect. Plant water status generally had higher total effects than RSM on leaf gas exchange, leaf area, and plant total biomass, and its effects on leaf gas exchange and leaf area were direct, while it affected plant total biomass major indirectly via leaf area (Supplementary Tables S5, S6).

### Critical RSM Thresholds of Maize Responding to Soil Water Deficit

**FIGURE 1:**
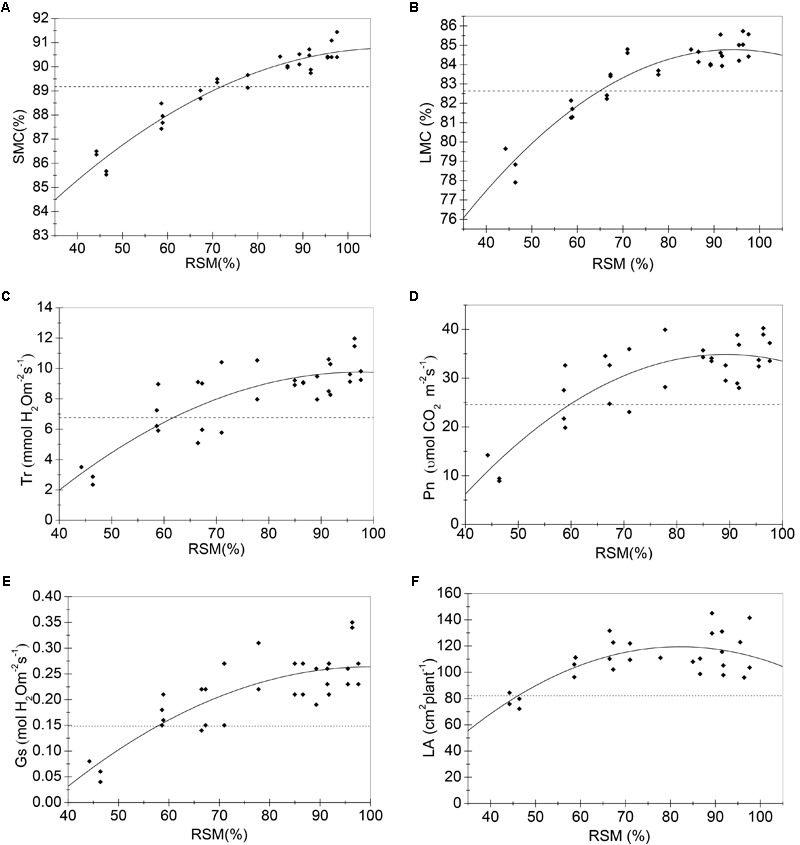
Regression models (solid lines) of RSM and sensitive growth characteristics of maize in the seedling stage in 2014. **(A)** Stem moisture content (%); **(B)** leaf moisture content (%); **(C)** transpiration rate (mmol H_2_O m^-2^s^-1^); **(D)** net photosynthetic rate (μmol CO_2_ m^-2^s^-1^); **(E)** stomatal conductance (mol H_2_O m^-2^s^-1^); **(F)** leaf area (cm^2^ plant^-1^). Closed squares refer to observed values; dashed lines refer to the tipping point of the growth characteristic.

**FIGURE 2:**
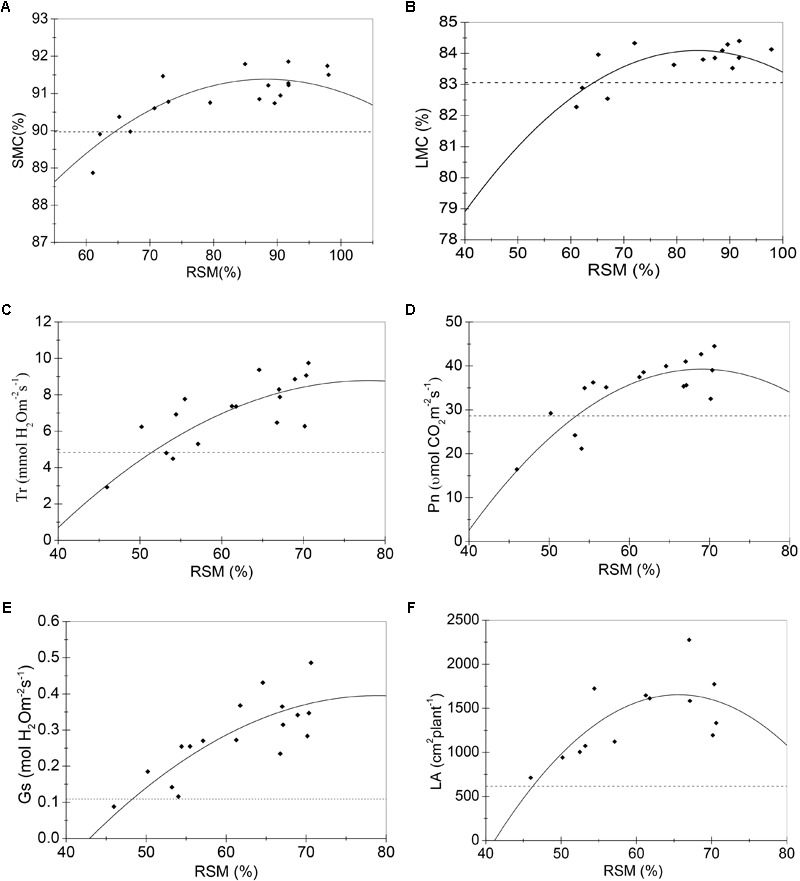
Regression model (solid lines) of RSM and sensitive growth characteristics of maize in the jointing stage in 2013. **(A)** Stem moisture content (%); **(B)** leaf moisture content (%); **(C)** transpiration rate (mmol H_2_O m^-2^s^-1^); **(D)** net photosynthetic rate (μmol CO_2_ m^-2^s^-1^); **(E)** stomatal conductance (mol H_2_O m^-2^s^-1^); **(F)** leaf area (cm^2^ plant^-1^). Closed squares refer to observed values; dashed lines refer to the tipping point of the growth characteristic.

Samples of all these sensitive growth characteristics distributed normally (*P* > 0.05) (**Table 4**), and they all decreased due to soil water stress (**Figures 1, 2**). Thus, their tipping points were identified by calculating the lower tolerance limits of the observed samples at 0.95 lower confidence level and including 95% percentile of the population (γ = 0.95, *P* = 95%) (Eqn. 4; **Tables 5, 6**). The values of critical RSM were further calculated. The critical RSM threshold was about 72% for SMC, about 65% for LMC, about 62, 60, 58, and 46% for Tr, net Pn, Gs, and leaf area, respectively, in seedling stage (**Figure 1** and **Table 5**). In jointing stage, the critical RSM was about 64% for both stem and LMC, about 53, 51, 48, and 46% for net Pn, Tr, Gs, and leaf area, respectively (**Figure 2** and **Table 6**).

**Table 4:** Shapiro–Wilk normal test for stress-free samples of maize growth characteristics.

Sensitive growth characteristics	Seedling stage (2014)		Jointing stage (2013)	
	Sample size	*P*	Sample size	*P*
SMC	12	0.451	15	0.696
LMC	22	0.923	12	0.727
Tr	18	0.321	12	0.982
Pn	18	0.356	12	0.440
Gs	18	0.152	12	0.798
LA	24	0.086	12	0.320

*SMC, stem moisture content (%); LMC, leaf moisture content (%); Pn, net photosynthetic rate (μmol CO_*2*_ m^-*2*^s^-*1*^); Gs, stomatal conductance (mol H_*2*_O m^-*2*^s^-*1*^); Tr, transpiration rate (mmol H_*2*_O m^-*2*^s^-*1*^); LA, leaf area (cm^*2*^ plant^-*1*^).*

**Table 5:** The critical thresholds and critical RSM of sensitive growth characteristics of maize in the seedling stage in 2014.

Sensitive growth characteristics	Parameters of regression models			*R*^2^	Critical thresholds	Critical RSM	
	a	b	c			Estimated values	95% confidence interval
SMC	*-*0.001	0.248	77.187	0.936	89.18	72%	[69%, 75%]
LMC	*-*0.002	0.468	62.734	0.886	82.63	65%	[62%, 68%]
Tr	*-*0.002	0.459	*-*12.595	0.686	6.76	62%	[56%, 67%]
Pn	*-*0.012	2.108	*-*59.197	0.711	24.60	60%	[55%, 64%]
Gs	*-*6.461E-5	0.013	*-*0.382	0.670	0.148	58%	[51%, 63%]
LA	*-*0.029	4.732	*-*74.822	0.495	82.1	46%	[39%, 52%]

*SMC, stem moisture content (%); LMC, leaf moisture content (%); Pn, net photosynthetic rate (μmol CO_*2*_ m^-*2*^s^-*1*^); Gs, stomatal conductance (mol H_*2*_O m^-*2*^s^-*1*^); Tr, transpiration rate (mmol H_*2*_O m^-*2*^s^-*1*^); LA, leaf area (cm^*2*^ plant^-*1*^)*.

**Table 6:** The critical thresholds and critical RSM of sensitive growth characteristics of maize in the jointing stage in 2013.

Sensitive growth characteristics	Parameters of regression models			*R*^2^ of regression models	Critical thresholds	Critical RSM	
	a	b	c			Estimated values	95% confidence interval
SMC	0.002	0.439	71.986	0.661	89.96	64%	[60%, 68%]
LMC	0.002	0.452	65.133	0.544	83.06	64%	[59%, 68%]
Tr	*-*0.006	0.871	-25.219	0.607	4.83	51%	[44%, 55%]
Pn	*-*0.044	6.014	-168.289	0.702	28.60	53%	[50%, 56%]
Gs	*-*3.053E-4	0.048	-1.505	0.719	0.109	48%	[40%, 52%]
LA	*-*2.769	363.320	–10264.4	0.530	616.9	46%	[41%, 50%]

*SMC, stem moisture content (%); LMC, leaf moisture content (%); Pn, net photosynthetic rate (μmol CO_*2*_ m^-*2*^s^-*1*^); Gs, stomatal conductance (mol H_*2*_O m^-*2*^s^-*1*^); Tr, transpiration rate (mmol H_*2*_O m^-*2*^s^-*1*^); LA, leaf area (cm^*2*^ plant^-*1*^).*

### Comparison on Meteorological Conditions

The three performed observation days were identically clear days, and their daily dynamics of temperature, total radiation, wind speed were quite close, except that VPD of July 9th, 2014 was relatively higher than that of July 31st, 2013 and August 8th, 2013 (Supplementary Figure S1).

## Discussion

### Critical Soil Water Thresholds of Maize

A threshold response of plant growth to soil or plant water status had been observed, that is, plant growth or physiological traits would remain almost constant until the water status was lower than a critical point (Blum, 1996; Sadras and Milroy, 1996; Ray et al., 2002; Novák, 2009; Gholipoor et al., 2012). In this study, maize growth characteristics appeared no significant difference among treatments with relatively higher soil moisture despite their soil water status were apparently different, however, they diverted significantly only in treatment(s) with lower soil moisture which was (were) below a certain level (**Tables 1–3**), implying their threshold responses to soil water status. Besides, the treatment differences were not identical among maize growth characteristics (**Tables 2, 3**), suggesting that they may have diverse critical soil moisture thresholds, namely, different sensitivities to soil water deficit. In both stages, growth characteristics related to plant water status (stem and LMC), leaf gas exchange (net Pn, Tr, and Gs), and leaf area varied at relatively higher soil moisture and thus were more sensitive to soil water deficit, while biomass-related characteristics appeared no significant difference under such wide soil water status (**Tables 2, 3**).

The results showed that the critical soil moisture thresholds were not identical either among growth characteristics or between stages (**Tables 5, 6**). In general, the critical soil moisture thresholds of maize growth characteristics in the seedling stage were higher than that in the jointing stage except that the critical soil moisture thresholds of leaf area were almost identical in both stages. The possible reason was that, in the seedling stage, compared to the jointing stage, the leaf water potential was higher (Sengupta and Majumder, 2014), the root was shallower, and leaves were smaller, which would result in weaker transpiration and poorer capability of soil water absorption (Kang and Liu, 1993). Thus, more abundant soil moisture was required to maintain high soil water potential to reduce the resistance of water absorption and to ensure water supply in the seedling stage (Ishida et al., 1992; Ying et al., 2015; Zhang et al., 2015).

Sadras and Milroy (1996) reviewed soil water thresholds (quantified by plant available water, PAW) of leaf expansion and leaf gas exchange among different species obtained under diverse experimental conditions. The average PAW threshold of leaf water potential was 0.61 ± 0.09, Gs was 0.37 ± 0.05, and leaf expansion was 0.56. Schmidt et al. (2001) found the tipping point of Gs of maize when PAW was 0.36. Ray et al. (2002) verified that the critical PAW of transpiration varied from 0.3 to 0.4 under different VPD. In our study, the PAW thresholds (converted from soil moisture) for SMC, LMC, Tr, net Pn, Gs, and leaf area were 0.58, 0.48, 0.43, 0.40, 0.37, and 0.19, respectively, in the seedling stage, while they were 0.46, 0.46, 0.27, 0.30, 0.22, and 0.19, respectively, in the jointing stage. The thresholds of Tr and Gs of this study were quite close to those from previous studies, in particular, those in the seedling stage almost coincided with the above results. However, these might merely coincidence, because these thresholds may vary dramatically among plant species, genotypes, phonology, soil property (i.e., soil texture and soil bulk density), root distribution, evaporative environment, growing conditions, and may also be influenced by regression models used and variations in determining the lower limit of PAW (Doorenbos and Kassam, 1980; Sadras and Milroy, 1996; Thompson et al., 2007; Casadebaig et al., 2008; Wu et al., 2011a; Schoppach and Sadok, 2012; Andrianasolo et al., 2016). For instance, PAW thresholds of maize could range from 0.27 to 0.85 for leaf expansion, and from 0.07 to 0.85 for leaf transpiration (Sadras and Milroy, 1996).

### Sequence of Changes in Maize Responding to Soil Water Deficit

The specific critical soil moisture thresholds of plant growth characteristics were not comparable among different studies, however, they may well reflect the sequence of changes in maize responding to soil water deficit (Sadras and Milroy, 1996). The critical RSM thresholds of maize showed the sequence of changes in maize as SMC > LMC > Tr > Pn > Gs > LA in seedling stage and SMC ≥ LMC > Pn > Tr > Gs > LA in jointing stage. Our study verified that plant water status was one of the earliest response to soil water deficit (Hsiao, 1973; Hsiao et al., 1976), followed by maize growth characteristics related to leaf water status, such as leaf water potential, leaf relative water content, and LMC which have long been used to indicate water stress (Hsiao, 1973; Hsiao et al., 1976; Ackerson et al., 1977; Kumar et al., 1994). Our study also showed that stem was more sensitive to water stress than leaf (Westgate and Boyer, 1985; McCutchan and Shackel, 1992; Shackel et al., 1997; Naor, 2000; Intrigliolo and Castel, 2006; Abrisqueta et al., 2015).

It has been found that stomata closed prior to decreases in Tr and Pn when confronted with water stress (Hsiao, 1973; Hsiao et al., 1976). However, in our study, the critical soil moisture of Gs in both stages were slightly lower than those of Tr and Pn. This was possibly because that Gs was quite sensitive to various factors more than soil water. For instance, stomatal interactions with environmental factors such as light and CO_2_ were complex and appeared to be mediated by several underlying processes (Hsiao, 1973). Increasing VPD between the leaf-air interface would also result in stomata closure regardless of soil water status (Farooq et al., 2009). Besides, hormones such as abscisic acid (ABA) would regulate stomata aperture during the early stage of drought, whereas factors such as leaf water potential, nutrition situation, pH of sap flow and farnesyltransferase activity could affect stomata’s sensitivity to ABA (Holbrook et al., 2002; Medrano et al., 2002). All these factors made Gs extremely variable, which resulted in greater variances within the samples that were subjected to tolerant limits calculation, yielding a broader tolerant limit and consequently lower critical RSM (Eqn. 4).

Expansion of mesophyll cells was thought to be even more sensitive to soil water deficit than the Gs and Pn (Hsiao, 1973; Hsiao et al., 1976; Fischer, 1980; Lambers et al., 2008). In contrast, our study found that leaf area responded at lower critical soil moisture than those of leaf gas exchange characteristics. In fact, they were not contradictory. Mesophyll cell expansion was a physiological process and thus responded instantly to water status, whereas leaf area was cumulative effects of soil water stress on mesophyll cell expansion as well as a result of adaptive growth of leaf under drought, which depended on not only expansion of all growing leaves, but also leaf number and the senescence of older leaves, consequently responding slower to drought (Reddy et al., 2003; Blum, 2011; Pinheiro and Chaves, 2011; Bodner et al., 2015).

Different sensitivities of maize growth characteristics to soil water deficit resulted from the way soil water affected them. The results of exploratory path analysis showed that soil water had the most significant total effects on plant water status, less on leaf gas exchange and leaf area. Besides, plant water status was directly affected by soil water, while leaf gas exchange and leaf area were more directly influenced by plant water status. Reductions in plant water status, especially leaf water status would give rise to a loss in turgor, which would on one hand reduce cell division and enlargement and consequently inhibit leaf expansion, on the other hand, lead to stomatal closure, consequently impeding CO_2_ influx and H_2_O outflux and thus leaf gas exchange (Hsiao, 1973; Blum, 2011; Pinheiro and Chaves, 2011; Sanders and Arndt, 2012). It implied that variations in leaf gas exchange and leaf area, to a greater extent, were secondary effects of soil water stress on plant water status, and thus they responded slower than plant water status to soil water deficit. The results of exploratory path analysis also showed that plant total biomass was directly influenced by leaf area, whereas soil moisture had no significantly direct effect on total biomass, nor had leaf water status, nor had leaf gas exchange. Plant total biomass was an accumulation of plant growth at various levels over a certain period, while soil moisture, plant water status, and leaf gas exchange were all instantaneous characteristics that only indicated water stress of the very time they were observed, so their effects could not be instantly detected from plant biomass. That’s why all these three characteristics appeared no significant direct effect on plant total biomass. However, long term effects of soil water deficit on leaf expansion and leaf gas exchange both appeared as a reduction in leaf area at the whole plant level, which led to decreased transpiration as well as lower intercepted radiation, and ultimately decreased biomass production (Reddy et al., 2003; Blum, 2011; Pinheiro and Chaves, 2011; Sanders and Arndt, 2012). It implies that changes in plant biomass were integrated results of all these secondary or even tertiary effects of soil water deficit in a longer term.

## Author Contributions

GZ, XM, and QH conceived and designed the research, analyzed the data, and wrote the manuscript. XM performed the experiments.

## Conflict of Interest Statement

The authors declare that the research was conducted in the absence of any commercial or financial relationships that could be construed as a potential conflict of interest.
