# Supplementary material for: Sequence of Changes in Maize Responding to Soil Water Deficit and Related Critical Thresholds
Source: Front Plant Sci. 2018 May 1;9:511. doi: 10.3389/fpls.2018.00511 (PMC5938554; doi:10.3389/fpls.2018.00511)

Supplementary material

**Table S1** The linear correlation coefficient between each sensitive growth characteristics and RSM of different depth in seedling stage in 2014

| RSM | LA | LMC | SMC | Pn | Tr | Gs |
| --- | --- | --- | --- | --- | --- | --- |
| 0-10 | 0.570^*^ | 0.855^**^ | 0.897^**^ | 0.846^**^ | 0.860^**^ | 0.846^**^ |
| 0-20 | 0.631^**^ | 0.893^**^ | 0.929^**^ | 0.893^**^ | 0.907^**^ | 0.884^**^ |
| 0-30 | **0.647^**^** | **0.906^**^** | **0.937^**^** | **0.904^**^** | **0.911^**^** | **0.886^**^** |
| 0-40 | 0.613^*^ | 0.899^**^ | 0.926^**^ | 0.894^**^ | 0.903^**^ | 0.886^**^ |
| 0-50 | 0.598^*^ | 0.883^**^ | 0.925^**^ | 0.874^**^ | 0.890^**^ | 0.877^**^ |
| 0-60 | 0.587^*^ | 0.869^**^ | 0.915^**^ | 0.858^**^ | 0.880^**^ | 0.871^**^ |
| 0-70 | 0.561^*^ | 0.841^**^ | 0.892^**^ | 0.826^**^ | 0.855^**^ | 0.852^**^ |
| 0-80 | 0.546^*^ | 0.816^**^ | 0.863^**^ | 0.793^**^ | 0.828^**^ | 0.832^**^ |
| 0-90 | 0.518^*^ | 0.773^**^ | 0.822^**^ | 0.744^**^ | 0.784^**^ | 0.790^**^ |

Note: RSM: relative soil moisture (%); SMC: stem moisture content (%); LMC: leaf moisture content (%);Pn: net photosynthetic rate(μmol CO_2_m^-2^s^-1^); Gs: stomatal conductance (mol H_2_Om^-2^s^-1^); Tr: transpiration rate (mmol H_2_Om^-2^s^-1^); LA: leaf area (cm^2^ plant^-1^).

**Table S2** The linear correlation coefficient between each sensitive growth characteristics and RSM of different depth in jointing stage on Jul. 31^th^, 2013

| RSM | LA | LMC | SMC | Pn | Tr | Gs |
| --- | --- | --- | --- | --- | --- | --- |
| 0-10 | 0.092 | 0.597^*^ | 0.745^**^ | 0.516^*^ | 0.592^*^ | 0.699^**^ |
| 0-20 | 0.098 | 0.598^*^ | 0.742^**^ | 0.475 | 0.573^*^ | 0.674^**^ |
| 0-30 | 0.222 | 0.700^**^ | 0.753^**^ | 0.570^*^ | 0.603^*^ | 0.723^**^ |
| 0-40 | 0.058 | 0.555^*^ | 0.731^**^ | 0.469 | 0.549^*^ | 0.665^**^ |
| 0-50 | 0.072 | 0.562^*^ | 0.743^**^ | 0.478 | 0.548^*^ | 0.667^**^ |

Note: RSM: relative soil moisture (%); SMC: stem moisture content (%); LMC: leaf moisture content (%); Pn: net photosynthetic rate (μmol CO_2_m^-2^s^-1^); Gs: stomatal conductance (mol H_2_Om^-2^s^-1^); Tr: transpiration rate (mmol H_2_Om^-2^s^-1^); LA: leaf area (cm^2^ plant^-1^).

**Table S3** The linear correlation coefficient between each sensitive growth characteristics and RSM of different depth in jointing stage on Aug. 8^th^, 2013

| RSM | LA | LMC | SMC | Pn | Tr | Gs |
| --- | --- | --- | --- | --- | --- | --- |
| 0-10 | 0.371 | 0.499 | 0.478 | 0.678^**^ | 0.683^**^ | 0.702^**^ |
| 0-20 | 0.554^*^ | 0.651^**^ | 0.593^*^ | 0.755^**^ | 0.753^**^ | 0.771^**^ |
| 0-30 | 0.694^*^ | 0.740^**^ | 0.675^**^ | 0.779^**^ | 0.781^**^ | 0.787^**^ |
| 0-40 | 0.693^**^ | 0.739^**^ | 0.663^**^ | 0.773^**^ | 0.772^**^ | 0.784^**^ |
| 0-50 | 0.656^*^ | 0.715^**^ | 0.654^**^ | 0.768^**^ | 0.761^**^ | 0.784^**^ |

Note: RSM: relative soil moisture (%); SMC: stem moisture content (%); LMC: leaf moisture content (%); Pn: net photosynthetic rate (μmol CO_2_m^-2^s^-1^); Gs: stomatal conductance (mol H_2_Om^-2^s^-1^); Tr: transpiration rate (mmol H_2_Om^-2^s^-1^); LA: leaf area (cm^2^ plant^-1^).

**Table S4** Model fit summary of the path analysis model

| Indexes | Criteria | Jul. 9^th^, 2014 | Jul. 31^th^, 2013 | Aug. 8^th^, 2013 |
| --- | --- | --- | --- | --- |
| χ^2^ | p>0.05 | **P=0.774(2.518)** | **P=0.115(10.225)** | **P=0.480(4.501)** |
| RMSEA | <0.10 | **0.000** | 0.204 | **0.000** |
| NFI | >0.90 | **0.984** | 0.857 | **0.926** |
| RFI | >0.90 | **0.969** | 0.761 | 0.852 |
| IFI | >0.90 | **1.016** | **0.935** | **1.009** |
| TLI | >0.90 | **1.033** | 0.885 | **1.020** |
| CFI | >0.90 | **1.000** | **0.931** | **1.000** |
| PCFI | >0.50 | **0.504** | **0.559** | **0.500** |
| CMIN/DF | <2.0 | **0.504** | **1.704** | **0.900** |
| AIC | Smaller than independent model and saturated model | **32.518<40.000**  **32.518<181.342** | **38.225<40.000**  **38.225<91.303** | **34.501<40.000**  **34.501<80.776** |

Note: bold folt means the model fit the criteria.

**Table S5** Standardized regression weights (R) and significant levels (P) of each effective path in the path analysis model

| Effective paths | Jul. 9^th^, 2014 | | Jul. 31^th^, 2013 | | Aug. 8^th^, 2013 | |
| --- | --- | --- | --- | --- | --- | --- |
|  | *R* | *P* | *R* | *P* | *R* | *P* |
| PWS ← RSM | 0.929 | ^***^ | 0.581 | ^**^ | 0.751 | ^***^ |
| LA ← PWS | 0.615 | ^***^ | 0.562 | ^**^ | 0.724 | ^***^ |
| LGE ← PWS | 0.856 | ^***^ | 0.616 | ^**^ | 0.366 | 0.063 |
| TB ← RSM | -0.489 | ^***^ | - | - | - | - |
| LGE ← RSM | - | - | - | - | 0.535 | ^**^ |
| TB ← LA | 1.005 | ^***^ | 0.950 | ^***^ | 0.621 | ^**^ |

***. means P < 0.001; **. means P < 0.01; *. means P < 0.05; -. means deleted paths due to insignificance (P > 0.05); ←leads from *independent variable* to *dependent* *variable*. PWS represents plant water status; RSM represents relative moisture of 0-30 cm depth; LA represents leaf area; LGE represents leaf gas exchange; TB represents plant total biomass.

**Table S6** Standardized total, direct, and indirect effects of independent variable on its dependent variable from each path of the path model

|  | Standardized Total Effects | | | Standardized Direct Effects | | | Standardized Indirect Effects | | | R^2^ |
| --- | --- | --- | --- | --- | --- | --- | --- | --- | --- | --- |
|  | RSM | PWS | LA | RSM | PWS | LA | PSM | PWS | LA |  |
| **Jul. 9^th^, 2014** | | | | | | | | | | |
| PWS | 0.929 | - | - | 0.929 | - | - | - | - | - | 0.86 |
| LA | 0.571 | 0.615 | - | - | 0.615 | - | 0.571 | - | - | 0.38 |
| Photo | 0.795 | 0.856 | - | - | 0.856 | - | 0.795 | - | - | 0.73 |
| TB | 0.120 | 0.129 | 1.005 | - | -0.489 | 1.005 | 0.120 | 0.617 | - | 0.64 |
| **Jul. 31^th^, 2013** | | | | | | | | | | |
| PWS | 0.581 | - | - | 0.581 | - | - | - | - | - | 0.34 |
| LA | 0.327 | 0.562 | - | - | 0.562 | - | 0.327 | - | - | 0.32 |
| Photo | 0.358 | 0.616 | - | - | 0.616 | - | 0.358 | - | - | 0.38 |
| TB | 0.310 | 0.534 | 0.950 | - | - | 0.950 | 0.310 | 0.534 | - | 0.90 |
| **Aug. 8^th^, 2013** | | | | | | | | | | |
| PWS | 0.751 | - | - | 0.751 | - | - | - | - | - | 0.56 |
| LA | 0.543 | 0.724 | - | - | 0.724 | - | 0.543 | - | - | 0.52 |
| Photo | 0.810 | 0.366 | - | 0.535 | 0.366 | - | 0.275 | - | - | 0.71 |
| TB | 0.337 | 0.449 | 0.621 | - | - | 0.621 | 0.337 | 0.449 | - | 0.38 |

Note: All the effects above were measured by standardized regression weight (R); -. means deleted paths due to insignificance; R^2^ is the squared multiple correlations of the variable. PWS represents plant water status; RSM represents relative moisture of 0-30 cm depth; LA represents leaf area; LGE represents leaf gas exchange; TB represents plant total biomass.

**Fig. S1** Daily dynamics of Meteorological elements. (A) Temperature (℃); (B) Vapor pressure deficit (VPD) (hPa); (C) Total radiation (MJ m^-2^ h^-1^); (D) Wind speed (m s^-1^). Circles refer to meteorological elements of July 7^th^ , 2014; Trangles refer to meteorological elements of July 31^th^ , 2013; Crosses refer to meteorological elements of August 8^th^ , 2013.

**Fig. S2** Exploratory path analysis model. (a) Initial path analysis model; (b) Final path analysis model based on data of Jul. 9th, 2014; (c) Final path analysis model based on data of Jul. 31th, 2013; (d) Final path analysis model based on data of Aug. 8th, 2013; → represents linear dependency of *dependent* *variable* on the *independent variable*, leading from *independent variable* to *dependent* *variable* and measured by standardized regression weight, which is listed just above the arrow; The value right above the variable represents its squared multiple correlation (R^2^), representing the proportion of its variance that is accounted for by all its independent variables; PWS represents plant water status; RSM represents relative moisture of 0-30 cm depth; LA represents leaf area; LGE represents leaf gas exchange; TB represents plant total biomass; ei (i = 1, 2, 3, 4) enclosed in a circle is the error term, representing random fluctuations and anything else on which the performance may depend but is not measured in this study.

**Fig. S1**


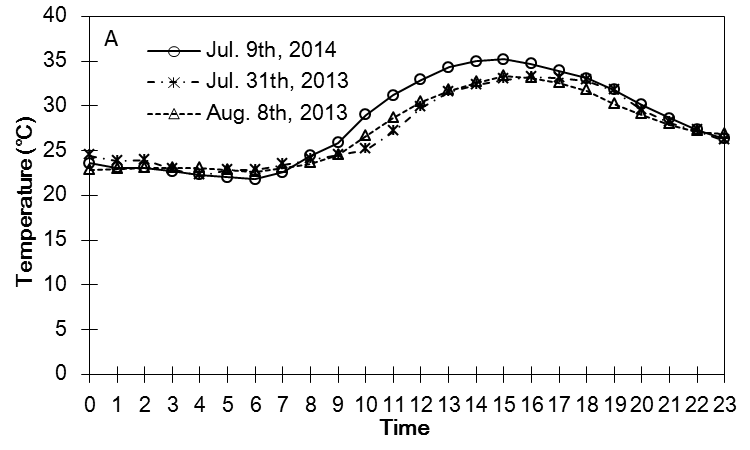

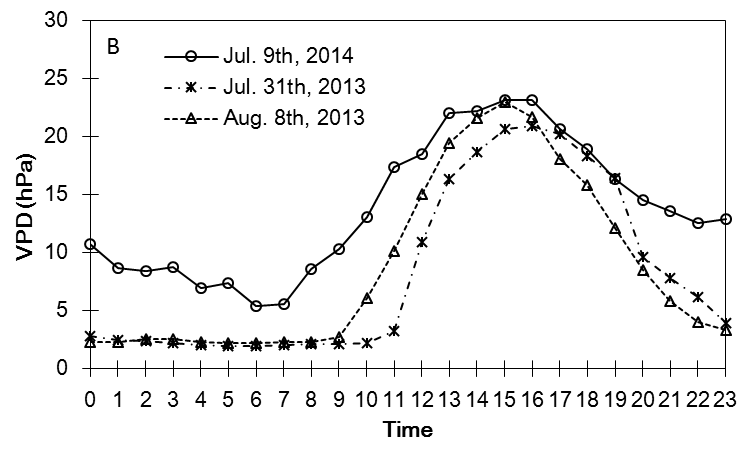


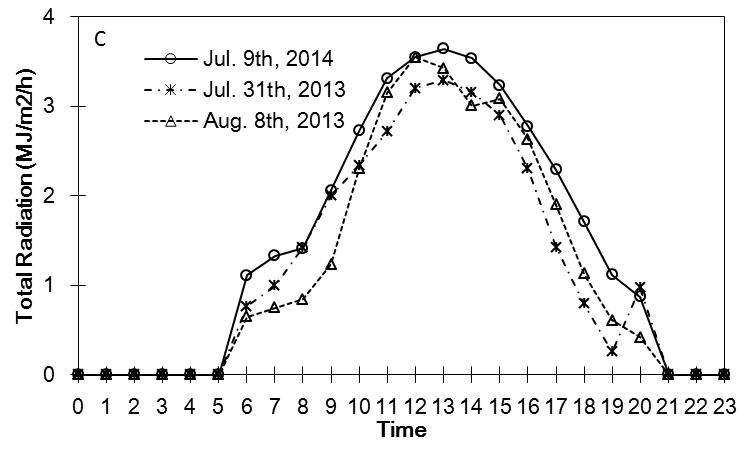

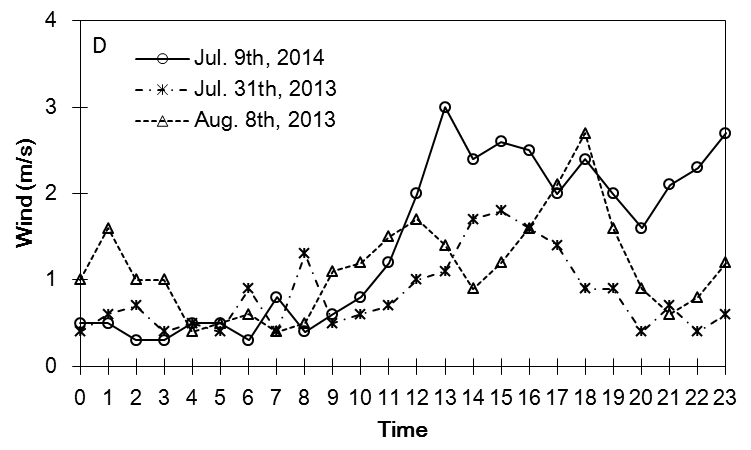


**Fig. S2**


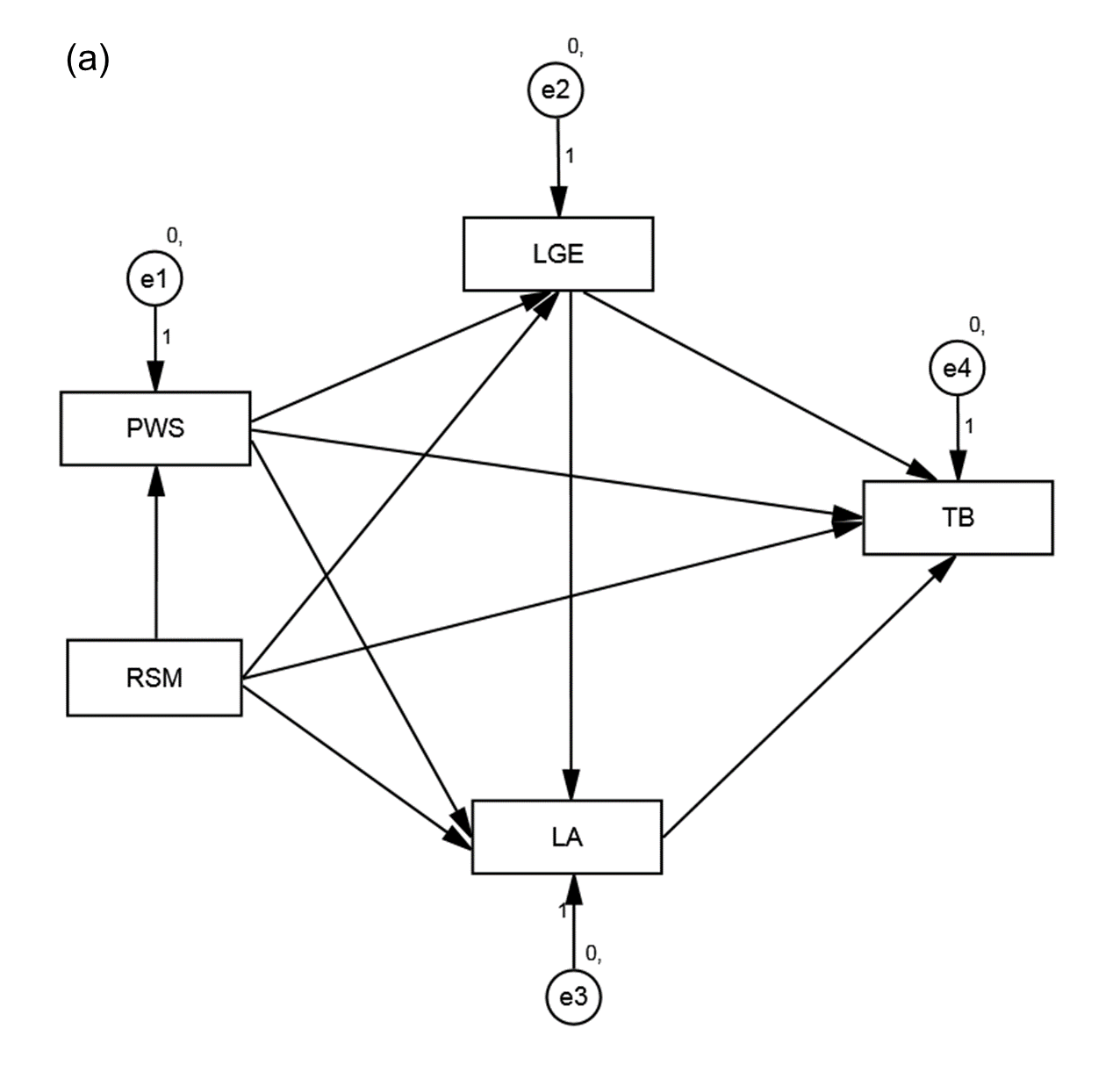

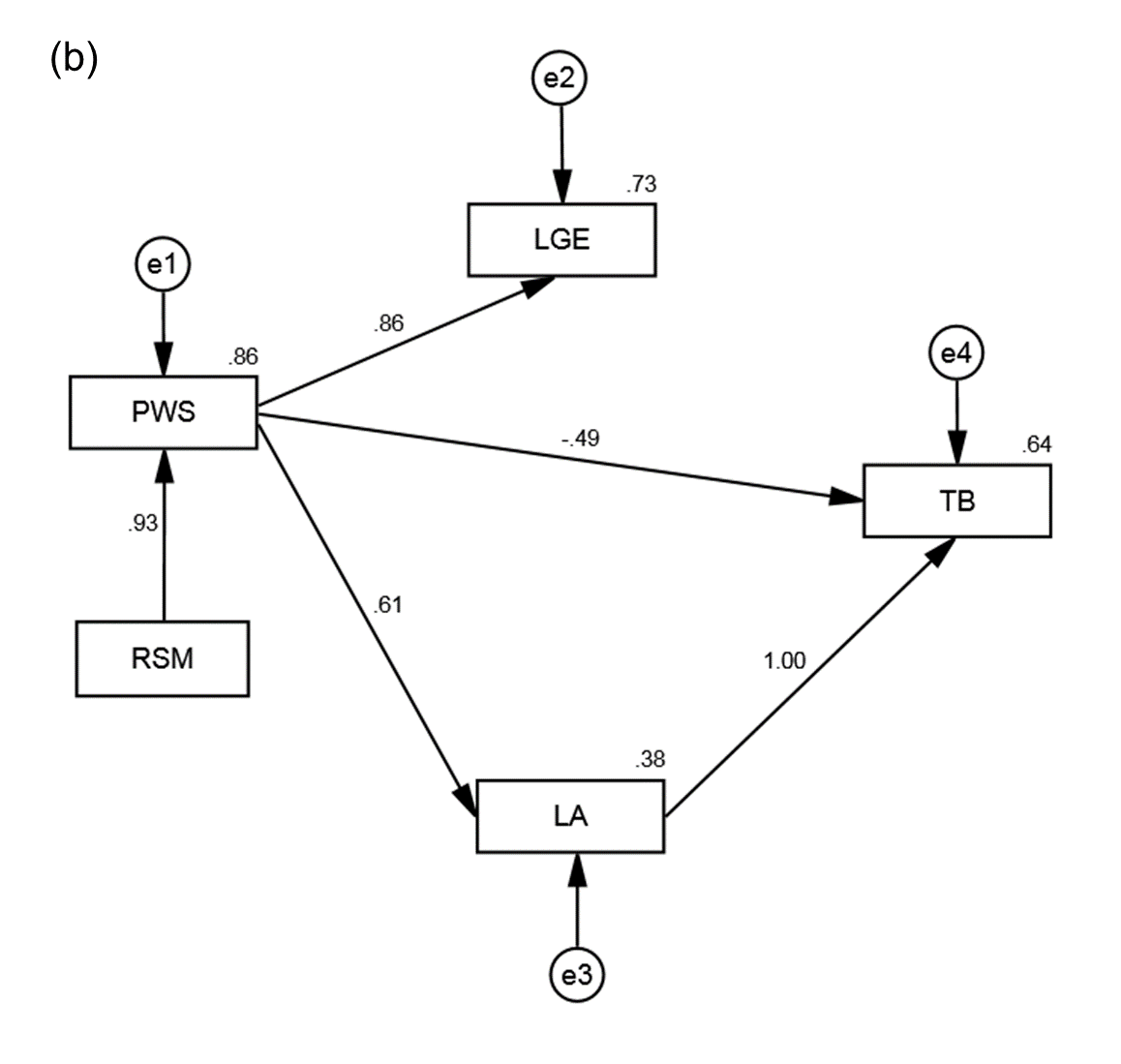

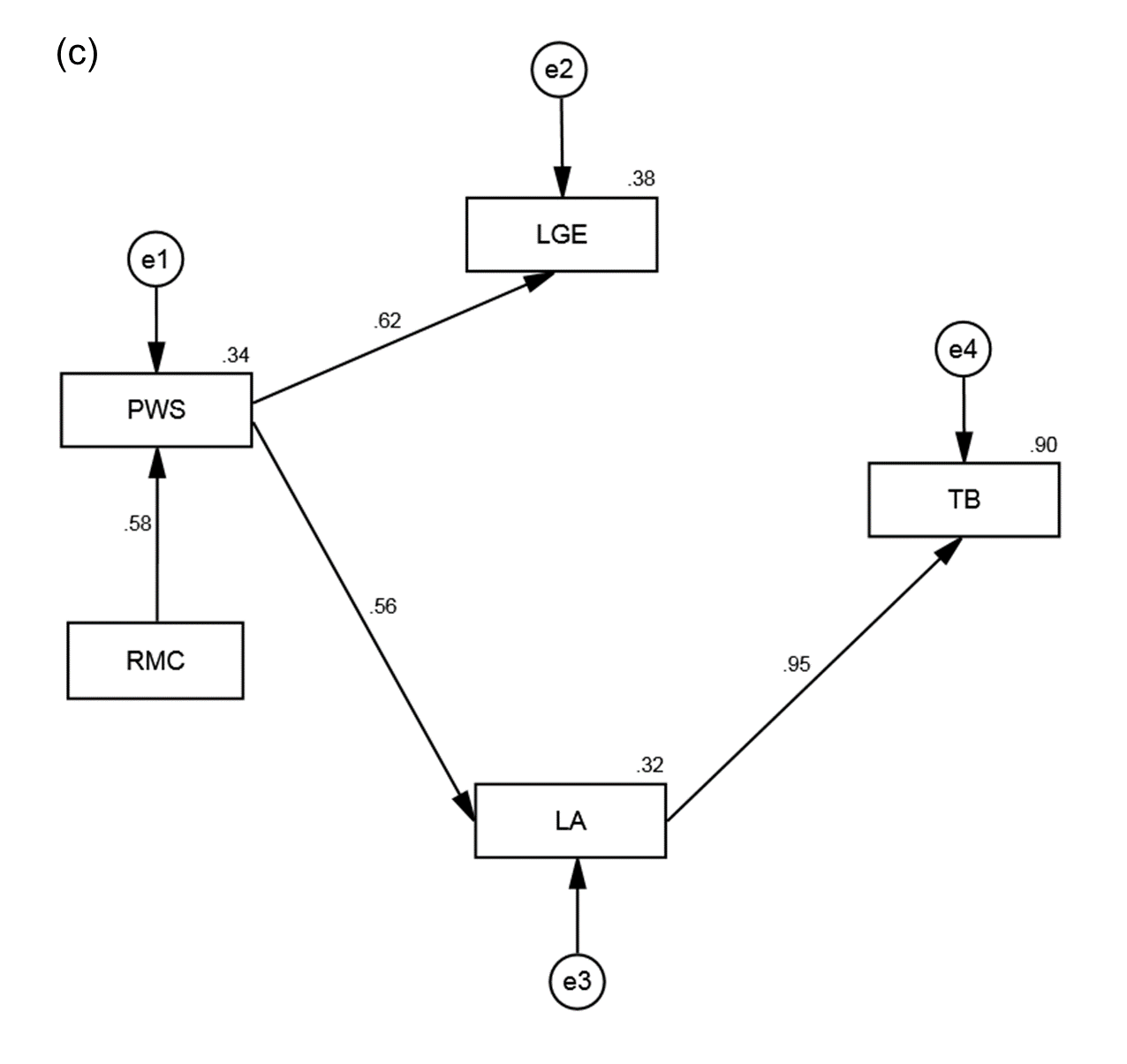

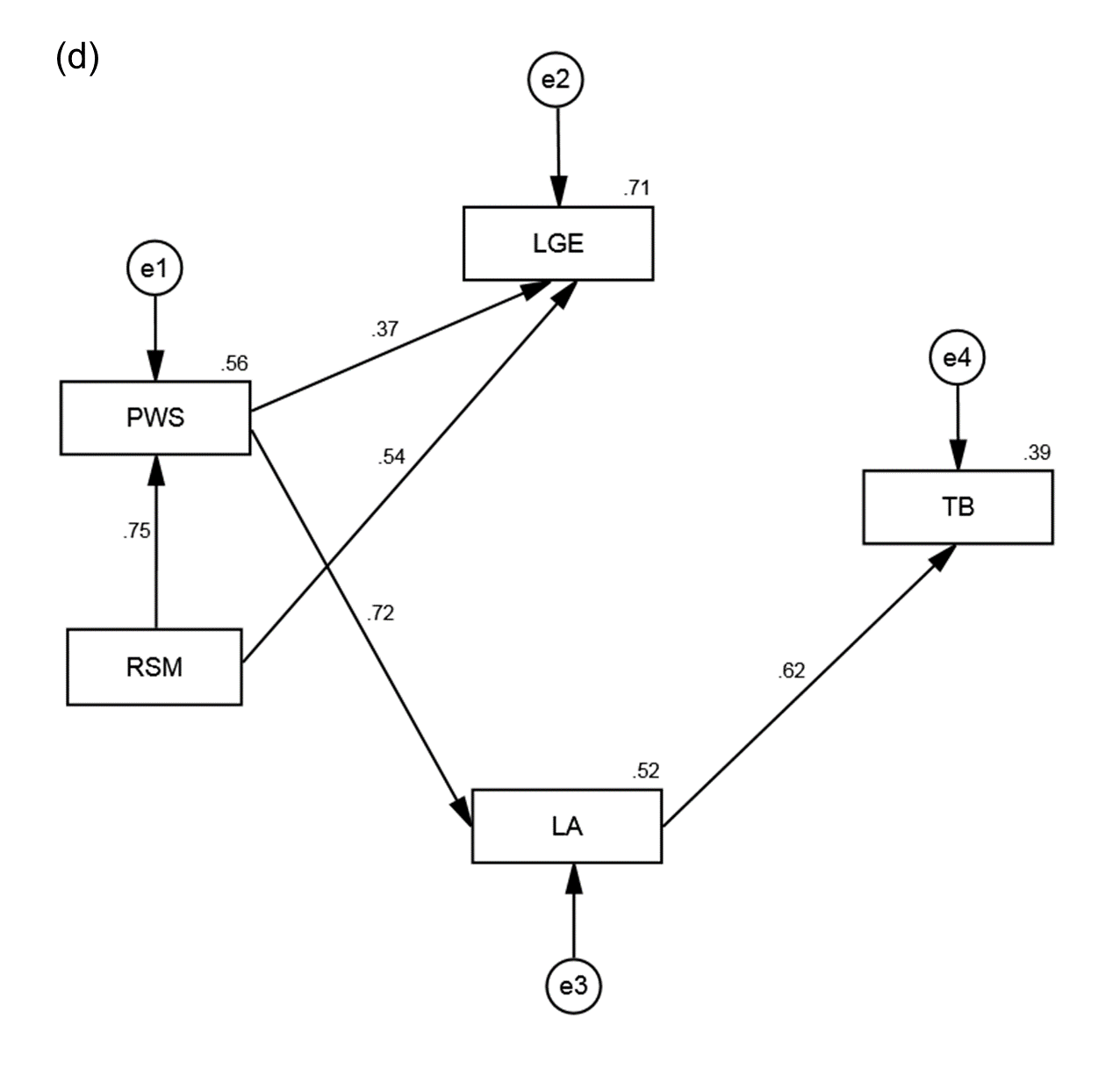

Supplement: Supplementary file 1 [file Data_Sheet_1.docx]
